# Macroporous Polymer Monoliths in Thin Layer Format

**DOI:** 10.3390/polym13071059

**Published:** 2021-03-27

**Authors:** Evgenia Korzhikova-Vlakh, Mariia Antipchik, Tatiana Tennikova

**Affiliations:** 1 Institute of Macromolecular Compounds, Russian Academy of Sciences, Bolshoy pr. 31, 199004 St. Petersburg, Russia; volokitinamariya@yandex.ru; 2 Institute of Chemistry, Saint-Petersburg State University, Unversitetskiy pr. 26, Petergof, 198584 St. Petersburg, Russia; t.tennikova@spbu.ru

**Keywords:** preparation of macroporous monoliths, polymer monolithic layers, thin-layer chromatography, microarray

## Abstract

Nowadays, macroporous polymer monoliths represent widely used stationary phases for a number of dynamic interphase mass exchange processes such as high-performance liquid chromatography, gas chromatography, electrochromatography, solid-phase extraction, and flow-through solid-state biocatalysis. This review represents the first summary in the field of current achievements on the preparation of macroporous polymer monolithic layers, as well as their application as solid phases for thin-layer chromatography and different kinds of microarray.

## 1. Introduction

Historically, the “monolithic era” began in the 1990s [1] from the development of rigid monoliths based on poly(glycidyl methacrylate-co-ethylene dimethacrylate) (poly(GMA-co-EDMA) [2] and polyacrylamide gel [3] monoliths as stationary phases for HPLC of proteins. These early efforts inspired a large number of scientists around the world to perform innovative researches, thereby rapidly advancing the field [4]. Today monolithic phases are obtained from synthetic (polymethacrylate, polyacrylamide, and polystyrene) [5,6,7] and natural (agarose and cellulose) polymers [8,9], or from inorganic substances [10]. Besides those, in the last decade, the hybrid organic–inorganic monoliths are extensively developed [11,12].

Among all types of monoliths, rigid macroporous polymer monoliths are one of the largest classes representing non-swellable highly crosslinked continuous materials containing interconnected macropores (d > 50 nm) [13,14,15]. In the late 1990s, encouraging results on chromatographic separations using rigid polymer monoliths inspired the industry. More than 20 years the rigid polymethacrylate and polystyrene monolithic stationary phases of various volume have been manufactured as CIM disks, columns, and tubes by BIA Separations (Ljubljana, Slovenia). Starting from 2021, BIA Separations became a division of Sartorius (Gottingen, Germany).

In comparison to diffusively controlled mass transfer in the particle-based sorbents, monoliths allow the realization of convectively-controlled interfacial mass transfer due to the high permeability of macroporous structure under increased flow rates. Mechanical and chemical stability of highly cross-linked polymer monoliths, as well as the ease of their preparation, are additional positive features of this kind of materials [16]. Rigid polymer monoliths can be synthesized in situ in chromatographic cartridge or capillary by thermo- or photoinduced polymerization of functional and crosslinking monomers in the presence of porogenic solvents [17,18]. Then the porogens are removed by washing, leaving voids in the polymer structure, which are macropores.

The emergence of interest in polymer monoliths is due to their effectiveness as stationary phases in various types of separation and analytical processes that are overviewed and discussed in some recent reviews [19,20,21,22]. At present, existing flow-through monoliths of various geometries (discs, rods, tubes, and capillary columns) with axial or radial flow have been developed for analytical and industrial applications [23,24,25,26]. A huge number of modern publications on the application of macroporous monoliths are devoted to the various flow-through techniques such as chromatographic separation of small molecules [27,28], synthetic polymers [29], and biological objects (proteins [30], peptides [31], oligosaccharides [32], DNA [33], viruses [34]), solid-phase extraction of wide range of compounds [35,36,37], and flow-through biocatalysis [38,39,40].

Despite the availability of commercial monolithic sorbents and wide application of flow-through monoliths, polymer monoliths are still the subject of extensive scientific investigation. The success of macroporous monoliths for application in flow-through processes gave a birth an idea to study this kind of materials as an analytical tool in a shape of thin layers [41,42]. This direction of monoliths development started in 2007 from the pioneering work of Bakry et al. who prepared the thin polymethacrylate monolithic layers and successfully applied them for thin-layer chromatography of peptides and proteins [41]. Simultaneously, Kalashnikova at el. illustrated the applicability of thin polymethacrylate-based monolithic layers for analysis of proteins in microarray format [42]. Both these trends in the development and utilization of monolithic matrices are quite young, but have already been presented in the literature in a number of works. In contrast to flow-through monoliths, broadly reviewed in the literature from different point of views [4,19,20,21,22,36,43,44], the summarization of achievements in the field of preparation and application of rigid macroporous monoliths in shape of thin layers have not been presented. Thus, the aim of this review is a first attempt to overview this topic.

## 2. Preparation of Macroporous Polymer Monolithic Layers

Currently, two main approaches for preparation of macroporous monolithic polymer layers have been described in the literature. The most used one is a traditional free radical polymerization in a mold with the use of organic solvents as porogens. Another one is a high-internal phase emulsion (HIPE) polymerization in a mold. In general, polymerization methods are similar to those used for preparation of flow-through monolithic rods. However, the preparation of macroporous monolithic layers has a number of features related to fabrication of a mold and its pre-polymerization treatment.

### 2.1. Modification of Supporting Surface

**Figure 1:**
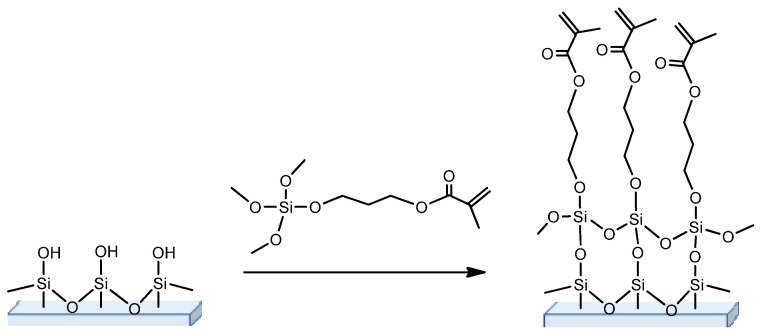
Scheme of the glass support silanization with the use of silane agent (usually TMSPMA) suitable for covalent attachment of macroporous polymer layer during its further polymerization.

One of the key obstacles of thin macroporous monolithic layers is their high fragility. In practice, this drawback is overcome by attaching the polymer layer to the inert support. Among the existed candidates, such as glass, metal or polymer plastic supports, the glass slides/plates are the most used due to their cheapness, inertness and the easy functionalization via the reaction with participation of silanol groups of glass support [45,46]. This technique for glass modification is simple, low cost and effective, and allows the introduction of any functionality of interests onto the glass surface [47]. The widely used silanization procedure, providing further covalent attachment of polymer monoliths to the glass, is based on the surface treatment with 3-(trimethoxysilyl)propyl methacrylate (TMSPMA) (Figure 1) whose double bond is able to participate in copolymerization with monomers during following polymerization. This silane agent possesses with moderate hydrophobicity and is suitable for copolymerization with acrylate, methacrylate and styrene type of monomers [41,48,49].

Before silanization, the glass surface must be activated with 0.2–1.0 M sodium hydroxide solution for 0.5–1 h to generate silanol groups. Currently, three different procedures of silanization have been corresponded to functionalize glass surface for further attachment of porous monolithic layer during its polymerization. Svec et al. used the procedure based on application of 20% TMSPMA solution in ethanol adjusted with acetic acid to pH 5 for 2 h [41] or later for 30 min [50]. Zheng et al. corresponded the application of 20% TMSPMA solution in ethanol overnight [51]. In our group, we used the silanization with 15% TMSPMA solution in toluene for 12–20 h [49,52,53]. Recently, we also replaced TMSPMA with acrylate derivative (TMSPA). The modification process was testified by measuring of contact angles between a water droplet and a surface. The unmodified glass surface exhibited 15.8 ± 0.2° whereas modified 66.4 ± 0.3° and 73.2 ± 0.8° for TMSPA and TMSPMA, respectively (unpublished data).

### 2.2. Fabrication of a Mold for Polymerization

**Figure 2:**
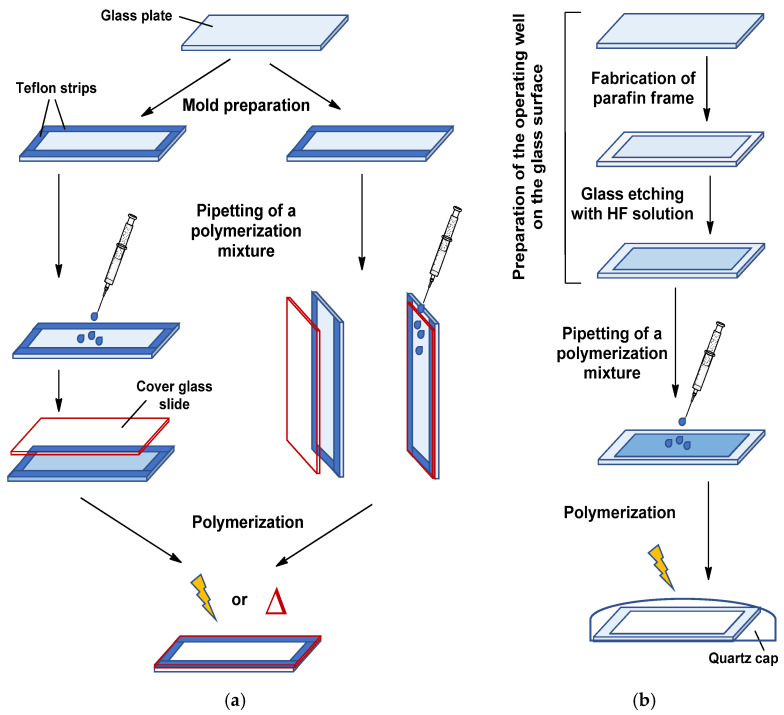
Schemes for the manufacture of polymer monolithic layers on the surface of the supporting glass plates: (**a**) Polymerization in a mold pre-formed from Teflon strips; (**b**) in situ polymerization in an operating well pre-formed by glass surface treatment. The covalent attachment of the polymer to the glass surface is achieved by the glass treatment with polymerizable silane agent (see Section 2.1 and Figure 1).

In contrast to monolithic rods, which are prepared inside chromatographic cartridges as molds, in the case of monolithic layers, the creation of a mold on the surface of the glass support is required for introduction of the polymerization mixture. In order to solve that task, two different techniques have been proposed and realized. The first approach was adopted from the technique of preparing polyacrylamide gels for gel-electrophoresis [54]. In particular, it is based on the formation of a mold from two glass plates clamped together and separated with Teflon strips (Figure 2a). The polymerization mixture is introduced by pipetting a polymerization mixture into formed mold or through a gap left on one side between two glass plates (Figure 2a). In this case, both photo- and thermo-initiated polymerization are possible [55,56,57]. The thickness of final polymer layer is pre-determined by the thickness of separating strips. In general, this approach for the preparation of monolithic layers is simple and requires only a good fixation of the suitable strips. The main problems that can be encountered when using this approach are the possible leakage of the polymerization mixture due to poor fixation of the mold and the difficulty in removing the cover glass after polymerization. Careless removal of the cover glass may cause damage to the layer.

Another technique is based on the manufacturing special operation wells on the glass surface via chemical etching of the glass. In particular, such wells can be fabricated by etching with hydrofluoric acid [58,59]. The application of paraffin mask for the part of glass surface allows keeping this part to be unetched. In this case, the thickness of final polymer layer is determined by the deepness of the manufactured well. The deepness of the well can be varied by the etching time or concentration of hydrofluoric acid. For instance, the etching of the standard microscopic glass with 11 M HF from 10 to 70 min provides the formation of the wells with deepness of 80 to 350 μm [60]. The alternative approach to chemical etching is a mechanical treatment of glass to manufacture the special wells for polymerization [60]. After manufacturing of the wells, the prepared slides are treated with 0.1 M NaOH under boiling for 30–40 min, washed with water until neutral pH, and dried. Polymerization mixture is added to the pre-silanized well, which then is covered with quartz cap and exposed under UV light to initiate polymerization [52,53,61] (Figure 2b). In comparison with the first approach for the preparation of monolithic layers, this one is more laborious in preparing the mold, but it is free from the disadvantages mentioned for the first method. At the same time, the utilized polymer layer can be mechanically removed and the glass mold with polished surface can be reused to synthesize a new polymer layer in it.

**Figure 3:**
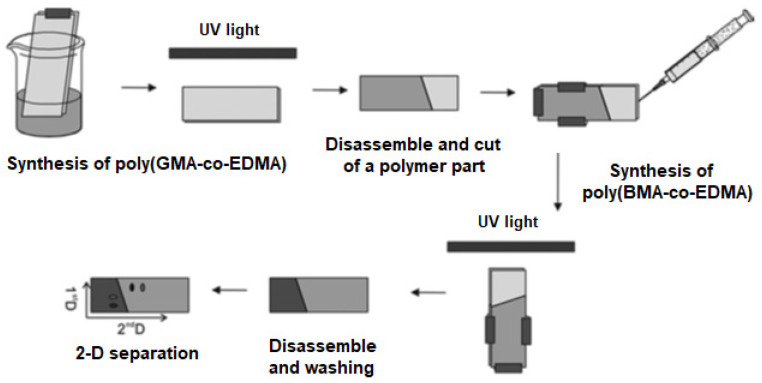
Scheme of preparation of the macroporous monolithic layer for 2-D TLC. Reprinted at modified form from [51] with permission of John Wiley and Sons.

The preparation of dual-phase polymer monolithic layers for 2-D TLC requires the application of some additional steps. For instance, Zheng et al. developed the method for preparation of dual monolithic phase consisting of two polymers with different hydrophilic-hydrophobic properties, namely poly(GMA-co-EDMA) and poly(BMA-co-EDMA) [51]. In that case, authors initially synthesized a layer of poly(GMA-co-EDMA) monolith and cut and removed a section of the synthesized polymer. Then, they filled the vacated area with second polymerization mixture containing BMA and EDMA and polymerized it to get the second polymer phase (Figure 3).

### 2.3. Polymerization and Post-Modification of Rigid Macroporous Polymer Layers

The polymerization mixtures used for the synthesis of rigid macroporous polymer monoliths in the shape of thin layers contain the same set of components as for preparation of rod-like monoliths [16,17,18]: (a) Functional and crosslinking monomer(s) to form polymer; (b) porogenic solvents to provide macroporous structure; and (c) initiator to produce initial free radicals for polymerization. Table 1 summarizes the monomers, initiators and porogens described for the synthesis of thin macroporous layers.

#### 2.3.1. Monomers

The first macroporous polymers, synthesized in the format of thin layer, were poly(BMA-co-EDMA) [41] and poly(GMA-co-EDMA) [42,55], which by that time had been well-known and widely used for hydrophobic and reversed-phase HPLC [67,68], affinity chromatography [69], and enzyme immobilization [70], respectively. As seen from Table 1, a wide range of monomers have been already utilized for the synthesis of monolithic layers; however, the most of them are still of polymethacrylate nature.

The functionality of monolithic layers can be achieved either by direct copolymerization of functional and cross-linking monomers or by post-modification of GMA-containing polymers. For instance, besides poly(GMA-co-EDMA), a number of macroporous monolithic layers with different properties have been synthesized in situ using such functional monomers as butyl methacrylate (BMA) [41], 2-hydroxyethyl methacrylate (HEMA) [70], 2-cyanoethyl methacrylate (CEMA) [71,72], 2-aminoethyl methacrylate (AEMA) [52,73], N-hydroxyphtalimide ester of acrylic acid (HPIEAA) [49], chloromethylstyrene (CMST) [57]. Besides EDMA, depending on a goal, more hydrophilic glycerol dimethacrylate (GDMA) [61] or di(ethylene glycol) dimethacrylate (DEGDMA) [53], or more hydrophobic divinylbenzene (DVB) [50,57] crosslinking agents were utilized.

For example, in work [71] the synthesis of CEMA-based macroporous monolithic layers was developed. In particular, such novel copolymers as poly(CEMA-co-EDMA) and poly(CEMA-co-GDMA) were synthesized. With the use of method of elemental analysis, it was found that the composition of copolymers was appeared to be very close to the monomer ratio used for polymerization.

Slabospitskaya et al. developed a number of macroporous monolithic layers based on poly(HPIEAA-co-GMA-co-EDMA) with different average pore size for protein microarray [49]. The polymer layers contained activated N-hydroxyphtalimide ester groups suitable for immobilization of amino-bearing bioligands. In comparison to well-known poly(GMA-co-EDMA), HPIEAA-monoliths provided much faster immobilization rate. The maximal immobilization capacity for HPIEAA-based terpolymer is achieved for 2 h at pH 7.4 [49], whereas the effective immobilization on GMA-monoliths requires about 16–20 h at more basic pH (8–9) [74].

In turn, Lv et al. reported the preparation of porous superhydrophobic monolithic layers for thin layer chromatography [57]. Monolithic poly(4-methylstyrene-co-chloromethylstyrene-co-divinylbenzene) (poly(MST-co-CMST-co-DVB)) layers of 50 μm thickness attached to glass supports have been prepared and additionally hyper crosslinked after polymerization. The presence of chloromethylstyrene units in the polymer allowed hyper crosslinking via a Friedel-Crafts alkylation reaction, and led to the formation of monoliths with much larger surface areas.

#### 2.3.2. Initiators

As in the preparation of flow-through monolithic columns, polymerization in thin layer starts after decomposition of a special compound (initiator) to free radicals as a result of external stimulus. Among the existed publications on the preparation of monolithic layers, one can find two main approaches for stimulation of initiator decomposition, i.e., thermo- [55,57] and photo-initiated ones [63,64,72]. Contrary to the preparation of monolithic columns, for which the main method is thermo-initiated decomposition of initiator [75,76,77], for thin layers the photo-initiated process is preferable (see Table 1). In general, UV-polymerization is limited by thickness of a mold and its transparency. In the case of flow-through monoliths, this technique is successfully realized for the synthesis of monoliths in quartz capillary for nanoHPLC [78] or capillary electrochromatography [79]. The dominance of UV-polymerization in the synthesis of thin layers is explained by the satisfaction to the above-mentioned requirements, on the one hand, and significantly shorter time of polymerization, on the other hand. In particular, depending on polymerization mixture composition and irradiation intensity the photo-initiated process takes 15–40 min, instead of several hours for thermo-initiated polymerization of the same polymerization system. For instance, Levkin et al. reported the preparation of thin layers based on poly(BMA-co-EDMA) and poly(ST-co-DVB) with the use of UV-initiated polymerization (2,2-dimethoxy-2-phenylacetophenone as initiator, λ = 254 nm) and thermo-initiated polymerization (azo-bis-isobutyronitrile as initiator, 70 °C) [55]. In first case, the polymerization was successfully completed after 15 min of irradiation, whereas the second technique required 24 h.

Besides 2,2-dimethoxy-2-phenylacetophenone as effective photo-sensitive initiator, 2-hydroxy-2-methylpropiophenone (Darocur-1173) and benzoin methyl ether (BME) have been found to be excellent initiators for the polymerization of methacrylate-type monomers. Fifteen-minute polymerization with the use of BME and twenty-minute with Darocur-1173 provided high polymer yield (~90%) and homogenous surface [66]. In comparison, benzophenone demonstrated low efficiency: polymer yield was only 73% after 80 min of polymerization. As to initiator amount, it was found that 0.8–1.0% from mass of monomers provided the maximum polymer yield.

#### 2.3.3. Porogens

A necessary condition for the formation of a porous structure is the presence in the polymerization mixture of the inert solvent(s) that acts as a porogenic agents. The key criterion for using substances as porogens is their poor compatibility with the resulting polymer [17,18]. The mechanism of the formation of the porous structure of monolithic carriers is well studied and described elsewhere [80,81]. Briefly, after the initiation of polymerization process, the polymer chains, formed in solution, precipitate as soon as they become insoluble in the reaction medium. Basically, phase separation occurs earlier if the porogens are “poor” solvents for the forming polymer. In turn, the “good” solvent or solvating diluent delays the phase separation process.

It is well known that in the synthesis of polymethacrylate and polystyrene monoliths such porogenic solvents as different alcohols work well [18,81]. For instance, these include cyclohexanol, 1-decanol, 1-dodecanol, 1-propanol, 1,4-butenediol, and PEGs [49,52,56,68]. For some hydrophilic polymers, such as poly(HEMA-co-EDMA/GDMA), the addition of small portions of toluene or solution of polystyrene (PS) in toluene appeared to be very effective to increase an average pore size [66]. However, an increase of the amount of polymeric component taken as porogen provided the increased viscosity and, in turn, favored to slow down the phase separation. As a result, a slight decrease of the average pore size and, simultaneously, an increase of specific surface area in the final monoliths were observed [66].

In comparison to flow-through monoliths applied in different dynamic methods, for thin monolithic layers the size of pores, e.g., 500 or 1500 nm, is not so crucial parameter since the mobile phase pumping is not supposed. Recently, Volokitina et al. studied the effect of average pore size on the efficiency of bioanalysis in the microarray format [53]. The monoliths based on poly(GMA-co-DEGDMA) with average pore size from ~400 to 1200 nm were studied in the process of protein analysis. It was found that affinity binding of protein from solution to immobilized probe was a bit faster for monoliths with smaller pore size during first 30 min. However, after that time no difference in binding efficiency was observed for monolith with different pore size. Similar effect was observed also for molecular recognition of phenylalanine derivatives by molecularly imprinted monolithic layers based on poly(AEMA-co-HEMA-co-EDMA) [73].

#### 2.3.4. Polymer Post-Modifications

Besides the direct copolymerization of functional monomers, a number of works has illustrated the functionalization of poly(GMA-co-EDMA) layers via surface grafting of hydrophilic [56], hydrophobic [56,63], and strong anionic [50,62] polymers. In particular, Urbanova et al. developed a method for preparation of hydrophobic-hydrophilic monolithic dual-phase plates by a two-step polymerization method [56]. For this, the 50 μm-thin poly(GMA-co-EDMA) layers attached to microscope glass plates were photografted with poly(ethylene glycol) methacrylate (polyPEGMA) to obtain superhydrophilic plates. The hydrophobicity was then formed by photografting of lauryl methacrylate. The exposure to UV light that initiates photografting was spatially controlled using a moving shutter that enabled forming of the diagonal gradient of hydrophobicity.

Han et al. reported the preparation of hydrophobic poly(BMA-*co*-EDMA) layers photografted through a mask with 2-acrylamido-2-methyl-1-propanesulfonic acid (AMMPSA) and 2-hydroxyethyl methacrylate (HEMA) for two-dimensional TLC assisted with ESI-MS detection [50]. In this case, the property of monolithic layers in certain zones were converted from hydrophobic to hydrophilic in order to provide further separations of biomolecules due to the ion-exchange and hydrophobic interactions with solid phase. Similar work was published by Woodward et al. [62] and devoted to the modification of poly(BMA-co-EDMA) with AMMPSA to prepare the sorbent for planar reverse-phased electrochromatography.

In the case of microarray preparation, it is necessary to functionalize surface with biological probes. The modification of polymer surface containing activated ester or epoxide groups can be done as one-step process. In turn, immobilization of biomolecules onto the hydrophilic surfaces as hydrolyzed poly(GMA-co-EDMA) or poly(HEMA-co-GDMA) monolithic layers requires the additional surface modification. In particular, Sinitsyna et al. demonstrated the successful protein immobilization using preliminary surface activation with carbonyldiimidazole [66] or disuccinimidyl carbonate [82]. In both cases, the successful protein immobilization can be achieved during 2–4 h at mild conditions (pH 7.4, 37 °C). In general, monolithic layers can be post-modified by the techniques already applied for flow-through polymer monoliths and summarized in the recent review [83].

### 2.4. PolyHIPE Monolithic Layers

Besides traditional monoliths formed by in situ polymerization of a mixture consisting of functional monomers, crosslinker, initiator and porogens, macroporous monoliths can be prepared by high internal phase emulsion (HIPE) polymerization technique. In the last case, polymerization of an emulsion with an internal phase volume fraction higher than 74% provides the formation of a porous polymer monolith with a network of large pores (up to 10 μm) pierced by smaller ones (~1–2 μm) [84,85]. For the water-in-oil emulsions water acts as the porogen and is dispersed in the organic phase, which contains polymerizable monomers and finally forms “continuous phase”. Water, or in other words, internal phase represents the discontinuous phase, which is removed after polymerization [85].

Due to high porosity and macroporous structure of polyHIPEs, this kind of monoliths have attracted attention as chromatographic stationary phases for different modes of HPLC and solid-phase extraction [85,86,87]. Recently, the preparation of polyHIPE monoliths for thin layer chromatography was reported by Yin et al. [48]. The authors developed the synthesis of poly(ST-co-BA-co-DVB) monolithic layers with the use of a water-in-oil HIPE technique. As a water phase they used 1.0 wt% CaCl_2_ aqueous solution while monomers, initiator and stabilizer (Span80) were mixed to prepare an oil phase. For polymerization, the viscous HIPE mixture was placed into the mold and covered with a glass plate to prevent the evaporation of components during following curing process. The polymerization was performed at 70 °C during 8 h. As a result, polyHIPE plates of 2 mm thickness were prepared and successfully applied in TLC analysis of extracts obtained from Chinese herbs.

**Figure 4:**
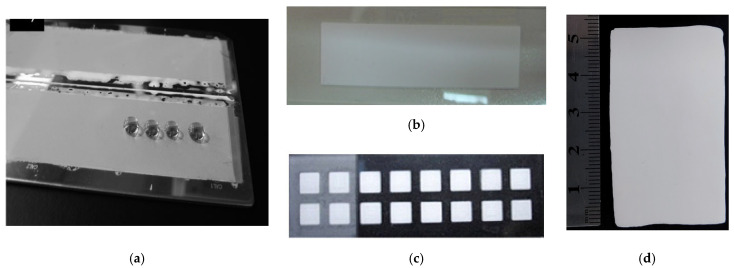
Images of thin monolithic layers prepared by different techniques: (**a**–**c**) traditional free radical polymerization of monomers in presence of porogenic solvents; (**d**)—HIPE polymerization. In (**a**,**d**) polymerization was carried out on the glass surface using a mold constructed from two glass slides separated with Teflon strips. In (**b**) polymerization was done in a well prepared via etching with HF solution while in (**c**) the polymer areas were synthesized in the small wells manufactured on the glass surface by mechanical treatment with thin milling cutter. Design of layers (**a**,**b**,**d**) is suitable for both TLC and microarray while (**c**) for microarray only. Image (**a**) reprinted from [55] with permission of Jhon Wiley and Sons. Images (**b**,**c**) taken from the unpublished collection of the authors of this review. Image (**d**) reprinted from [48] (RSC Open Access).

**Figure 5:**
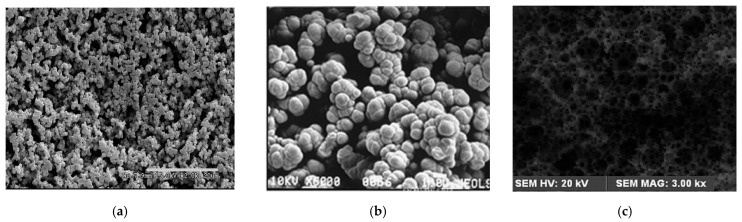
SEM images of macroporous polymer monolithic layers: (**a**) poly(BMA-co-EDMA) [41], (**b**) poly(HEMA-co-GDMA) [66] and (**c**) poly(ST-co-BA-co-DVB) (obtained by HIPE technique) [48]. Images (**a**,**b**) reprinted with permissions of ACS [41] and Elsevier [71]. Image (**c**) reprinted from [48] (RSC Open Access).

Thus, macroporous polymer monolithic layers can be easily prepared in situ by both traditional polymerization of monomers in a presence of porogenic solvents or by HIPE technique. Images of thin monolithic layers prepared by different techniques and their morphology are presented in Figure 4 and Figure 5, respectively.

## 3. Application in Thin-Layer Chromatography

A selection of a suitable stationary phase for TLC is one of the key factors for the successful separation of a mixture of components. Chromatographic thin layers can be formed from a variety of organic and inorganic sorbents, and various plates are commercially available [88,89]. The stationary phase in TLC is usually the porous unmodified or modified inorganic particles (silica gel) deposited in a thin layer on a plate. The sorbent granule size and particle size distribution determine the separation efficiency. The flow of the eluent is carried out due to capillary forces that depend on the diameter of the interparticle channels.

In the beginning of 21st century, monolithic silica materials in the form of thin layers were developed and proposed for TLC [90,91,92]. This inorganic phase is characterized by a bimodal pore size distribution, which means that its skeleton consists of large transport macropores 1–2 μm in diameter and a network of mesopores several nm in size. The advantage of such a solid phase in TLC has been proven by a significant increase in adsorption capacity, speed, and chromatographic efficiency [90,93,94]. The monolithic silica-based layers (UTLC plates) have been commercialized by Merck-Millipore [95].

The development of polymeric monoliths for TLC have been started in 2007 from the pioneering work published by Bakry et al. [41]. Below, one can find a summary on application of polymer monolithic layers in order to separate the substances of various classes of compounds using different TLC techniques. In general, the technique of TLC implementation with the use of monolithic layers is similar to traditional TLC. Since the macroporous structure of the polymer monolithic layer is pierced with a network of interconnected capillaries, the flow of the mobile phase through the solid phase occurs due to capillary forces.

### 3.1. Separation of Low-Molecular Compounds

Monolithic sorbents based on macroporous poly(BMA-co-EDMA) and poly(GMA-co-EDMA), known to be efficient in HPLC separations, as well as novel poly(AEMA-co-HEMA-co-EDMA) were prepared as thin layers by Maksimova et al. and studied in the separations of low-molecular weight dyes and DNP amino acids [52]. Tested dyes, namely *p*-aminoazobenzene, *p*-aminoazotoluene, and methyl red, belonged to the class of aromatic azo compounds and differed only by the nature of the substituent groups in the aromatic ring. It was shown that the separation of the mixture of dyes on poly(GMA-co-EDMA) layers was poor. Changing the solvent ratio of the mobile phase did not lead to an increase in selectivity. In turn, the application of more hydrophobic poly(BMA-co-EDMA) layers provided a separation of the components with good resolution within 7 min in ethyl acetate/ethanol/water = 7/8/7.5 (*v/v/v*) as a mobile phase. In contrast to aromatic azo compounds separated by reversed-phase mechanism of TLC, the separation of DNP-leucine, DNP-aspartic acid, DNP-tryptophan and DNP-alanine was successfully performed at normal phase TLC using poly(AEMA-co-HEMA-co-EDMA) layers and the mobile phase consisted of a mixture of hexane/chloroform/acetic acid = 33/64/3 (*v/v/v*). Good separation of the selected substances within 7 min has been demonstrated. *R_f_* values increased in the range DNP-aspartic acid, DNP-tryptophan, DNP-alanine and DNP-leucine, while the polarity increased in the opposite order.

Recently, Yin et al. reported the application of macroporous polyHIPE monolithic layers based on poly(ST-co-BA-co-DVB) as a stationary phase for TLC to identify Chinese herbal medicinal components (mostly anthraquinones) [48]. The resolution of spots for compounds separated on poly(ST-co-BA-co-DVB) layers was better comparing to that observed for only styrene-based layers. Moreover, polyHIPE monolithic layers found to be reusable stationary phases for TLC with high performance.

### 3.2. Separation of Peptides and Proteins

In the pioneering work on application of macroporous monoliths for TLC, Bakry et al. reported the preparation and application of macroporous poly(BMA-co-EDMA) layers for the separation of peptides and proteins with MALDI-TOF-MS detection [41]. Three peptides, namely [Sar^1^, Ile^8^]-angiotensin II, angiotensin II, and neurotensin, were labeled with fluorescamine and separated using 0.1% TFA in 45% aqueous acetonitrile (*v*/*v*) as a mobile phase. The location of the separated peptides was first detected visually using a UV lamp. The results showed a good separation of all three spotted peptides. The *R_f_* values were found to be 0.52, 0.41, and 0.30 for [Sar^1^, Ile^8^]-angiotensin II, angiotensin II, and neurotensin, respectively. Additionally, MALDI-TOF-MS analysis was carried out with the use of R-cyano-4-hydroxycinnamic acid as a matrix improving the ionization efficiency. Spraying protocol for matrix application was preferable than spotting one due to the absence of lateral migration of compounds in the thin layer detected for the second method.

The same monolithic layers were tested also for the separation of standard protein mixture containing four proteins. In the first series of experiments, these proteins were labeled with fluorescamine for UV-detection. The best solvent system for the protein mixture included 0.1% TFA in 55% aqueous acetonitrile (*v/v*). The *R_f_* values were found to be 0.836, 0.362, 0.286, and 0.205 for insulin, cytochrome c, lysozyme, and myoglobin, respectively. Then, the separation of unlabeled compounds was repeated on a monolithic layer attached to the MALDI target, and the spots were analyzed with MALDI-TOF-MS using sinapinic acid as a matrix sprayed over the plate. All nonlabelled proteins were successfully detected.

**Figure 6:**
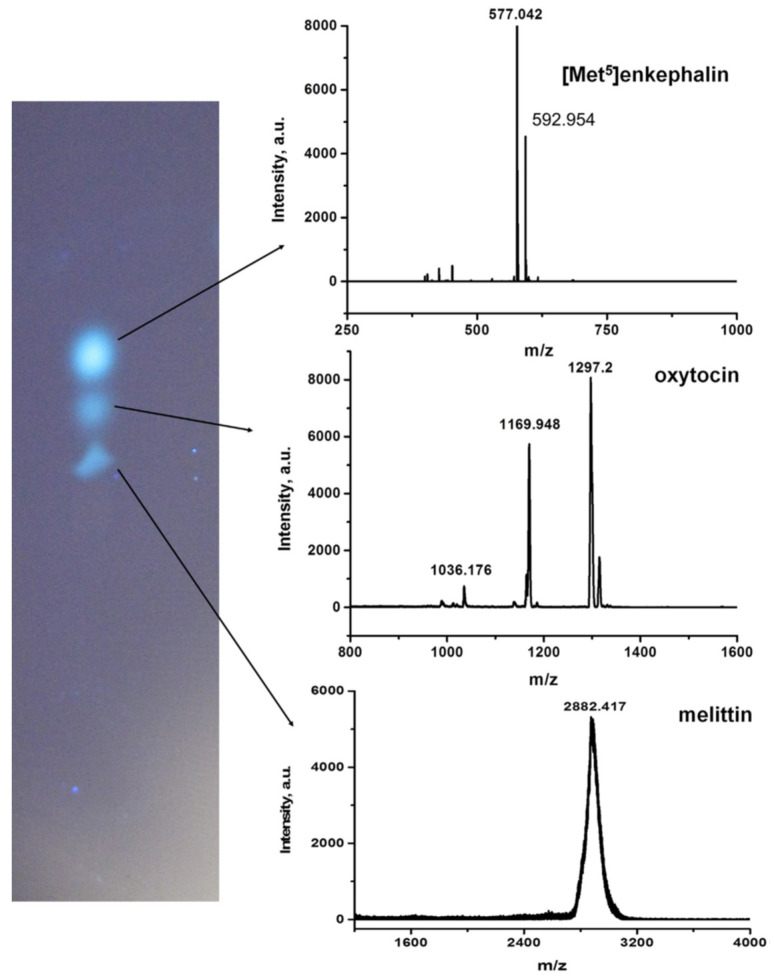
TLC separation of a mixture of peptides labeled with fluorescamine and MALDI-TOF-MS spectra obtained from the spots using cyano-4-hydroxycinnamic acid matrix. Reprinted with permission of Elsevier from [57].

Later, Lv et al. developed the porous hyper crosslinking monolithic layers [57] based on poly(MST-co-CHST-co-DVB). The TLC performance of these thin layers was first tested using a mixture of three peptides (melittin, [Met^5^]enkephalin and oxytocin) labeled with fluorescamine. The separation of all peptides was achieved in 15 min. In comparison to monolithic poly(BMA-co-EDMA) layers, the styrene-based sorbents possessed with higher hydrophobicity, and as a result, demonstrated better separation and slower migration even when the mobile phase contained the higher percentage of acetonitrile. Besides UV visualization, MALDI-TOF-MS after spraying the plate with a solution of R-cyano-4-hydroxycinnamic acid was also applied. The MS spectra with a good signal-to-noise ratio were obtained from the separated spots (Figure 6). Using similar conditions, three labeled proteins (ribonuclease A, lysozyme, and myoglobin) were separated in 15 min to distinct spots. The elution order agreed with the hydrophobicity of the individual proteins. Mass spectra confirmed the identity of the labeled proteins.

### 3.3. Separation of Synthetic Polymers

**Figure 7:**
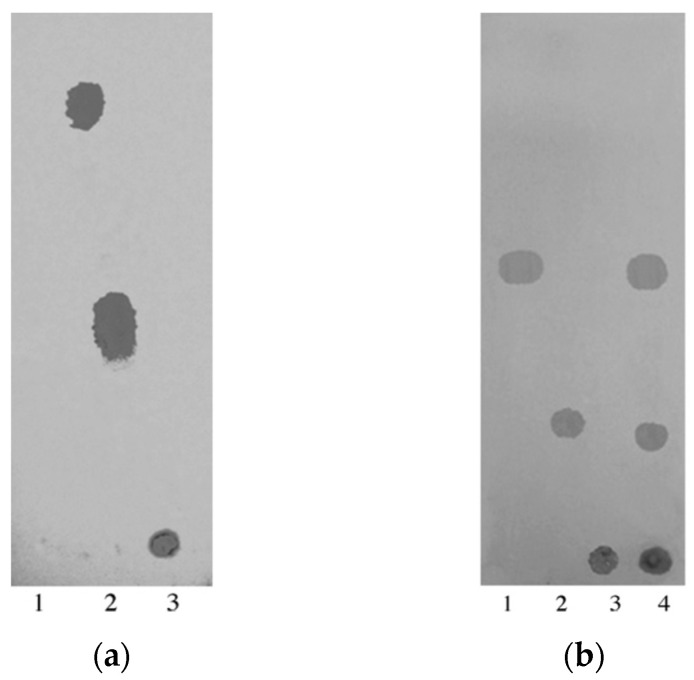
TLC separation of polymers: (**a**) poly(GMA-co-EDMA) layer; samples: 1–3—PVPs with M_w_ = 14,400; 94,700 and 1,065,000, respectively; mobile phase: water–isopropanol = 90:10 (*v/v*); (**b**) poly(BMA-co-EDMA) layer; samples: 1–3—PSs with M_w_ = 154,000; 500,000 and 960,000, respectively, 4—mixture of PSs; mobile phase: AcN–THF = 5:5 (*v/v*). Reprinted with permission of Elsevier from [52].

In contrast to the separation of small molecules, chromatography of polymers is a more complicated task. The multivariate chemical structure affecting the conformation of the polymer chain and its ability to be adsorbed on the sorbent surface, as well as the low diffusion coefficients play a key role in the separation process. Nevertheless, the correct selection of stationary phase and mobile phase can provide successful separation of synthetic polymers having the same nature but differed with their molecular weights. In particular, Maksimova et al. demonstrated the successful separation of three poly-N-vinylpyrrolidones (PVPs) with different molecular weights (M_w_ = 14,400; 94,700 and 1,065,000) using macroporous poly(GMA-co-EDMA) layers [52]. The separation PVP samples was not achieved when single-component eluent was used. However, an appropriate selectivity was achieved with the use of a mixture of water with organic solvent (Figure 7a). It was found that for separation of PVPs on poly(GMA-co-EDMA) layers, the displacement ability of organic solvents corresponded to the following order: methanol < acetonitrile < ethanol < isopropanol. Thus, the chromatography of PVPs on poly(GMA-co-EDMA) layers occurs according to the reversed-phase mechanism.

In the same work, authors also developed the protocols for the separation of polystyrenes (PSs) with different molecular weights (M_w_ = 154,000; 500,000 and 960,000) via reversed-phase mechanism on poly(BMA-co-EDMA) and normal-phase mechanism on poly(CEMA-co-HEMA-co-EDMA) layers using acetonitrile-tetrahydrofuran (Figure 7b) and n-hexane–tetrahydrofuran as mobile phases, respectively [52].

### 3.4. 2-D TLC

In a simple variant of two-dimensional (2-D) TLC, the separation is performed in two directions via 90° rotating the layer and using another mobile phase [96]. In more complicated approaches the layers combining two different chemistries are applied. In the field of macroporous monolithic layers for 2-D TLC, the development of superhydrophobic polymer layers with a photopatterned hydrophilic channel or superhydrophilic polymer with a diagonal gradient of hydrophobicity for separation of peptides was reported by Svec’s group [50,56].

In the first case, porous superhydrophobic monolithic layers were prepared via photografting of poly(BMA-co-EDMA) with AMMPSA and HEMA through a mask with an opening defining the channel [50]. Since the selected peptides, namely leucine enkephalin, bradykinin, angiotensin II, and Val-Tyr-Val, were differed both in pI value and in hydrophobicity, the polymer layers were designed to provide separation via ion-exchange and hydrophobic interactions. First, the separation of peptides was carried out by ion-exchange mechanism within the hydrophilic channel using a mobile phase of 30% acetonitrile in aqueous 0.2 M ammonium acetate at pH 7.0 (*v/v*). Then, the dried plate was turned by 90° and the separation in the second dimension by reversed-phase mechanism was performed in a chamber using 0.1% trifluoroacetic acid in 40% aqueous acetonitrile (*v/v*). The results of separation for fluorescamine-labeled peptides were evaluated by UV detection while the native peptides were analyzed using a desorption electrospray ionization mass spectrometry (DESI-MS). It was found that spectra after 2-D separation were significantly cleaner that indicates the improved resolution after separation in the second dimension.

In the second case, hydrolyzed porous poly(GMA-co-EDMA) layers were photografted with PEGMA to obtain superhydrophilic plates and then with the use of specially moved mask a diagonal gradient of hydrophobicity have been created via photografting of LMA [56]. The plates obtained were utilized for a rapid 2-D separation of a mixture of four model peptides (Gly-Tyr, Val-Tyr-Val, leucine enkephalin, and oxytocin) using different mobile phases in each direction. The separated peptides were successfully detected with the use of MALDI mass spectrometry.

**Figure 8:**
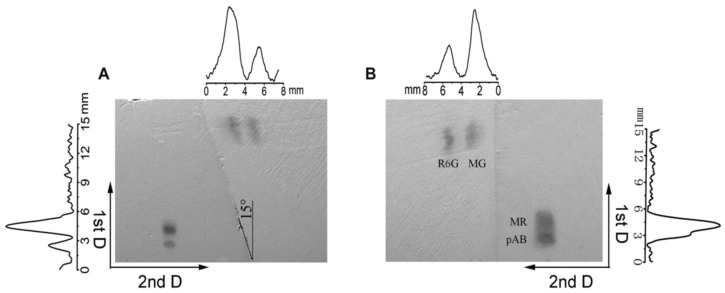
2-D TLC separation of four dyes with the use of dual-phase layer consisting of hydrophilic hydrolyzed poly(GMA-co-EDMA) and hydrophobic poly(BMA-co-EDMA) parts: (**A**) Slant boundary 15°, (**B**) slant boundary 0°. Mobile phase: (First dimension) ethyl acetate/ethanol/water 4:5:9 *v/v/v*, (second dimension) 10 mM NaCl methanol solution. Reprinted with permission of Jhon Wiley and Sons from [51].

The combination of two polymers with different nature, namely poly(BMA-co-EDMA) and hydrolyzed poly(GMA-co-EDMA), was realized by Zheng et al. [51]. Separation using hydrophobic poly(BMA-co-EDMA) can be achieved by reversed-phase mode of TLC, whereas hydrolyzed poly(GMA-co-EDMA) allows the realization of hydrophilic separation mode. The developed hydrophobic-hydrophilic monolithic porous polymer layer was used for 2-D separation of four dyes: *p*-amino-azo-benzene (pAB), methyl red (MR), malachite green (MG) and rhodamine R6G (R6G). Separation in the first dimension was performed in 3 min by using the ethyl acetate/ethanol/water = 4:5:9 (*v/v/v*) as the mobile phase. Then the dried layer was rotated for the separation in second dimension in 10 mM NaCl methanol solution as the mobile phase (Figure 8).

## 4. Application in Microarray

As mentioned above, macroporous monolithic layers have been successfully used in various modes of TLC. However, the characteristics of this kind of materials favored to their use in microarray technology. One of the main advantages of the macroporous monoliths is their structure, which ensures the unhindered penetration of large biomolecules (proteins, DNA) [30,33] and particles (viruses, cells) [34] into the pore space and perform highly sensitive bioanalysis.

Microarray is a modern tool for parallel analysis and screening of multicomponent biological samples [97,98]. This technique is based on the specific recognition of biomolecule, presenting in the complex biological mixture, by complementary bioligand immobilized on the solid support and called also as probe. The efficiency of interphase biorecognition directly depends on the properties of solid support and probe immobilization approach. An “ideal” solid support must meet the following criteria: (a) be biomolecule-friendly; (b) have high chemical stability under analytical conditions; (c) provide stable attachment of the probe to a solid surface; (d) have low autofluorescence and (e) provide good spot uniformity.

Today the supports for microarray can be classified into two groups. The first one represents so-called two-dimensional (2-D) supports. In this case, the biomolecule of interest reacts with a probe monolayer on the surface. For example, functionalized glass slides or non-porous synthetic polymer supports are widely used as 2-D supports for microarray [99,100]. The second group is based on three-dimensional matrices and includes polyacrylamide or polysaccharide gels, nitrocellulose membranes, etc. [101,102,103]. Three-dimensional supports are usually formed on a rigid inert surface, for example, glass or plastic layers [102,103]. In comparison to 2-D supports, three-dimensional microarrays have a much larger surface area, which allows immobilization of higher amounts of probe and, therefore, detecting higher signal intensities after affinity binding of labeled target biomolecule [104]. Moreover, three-dimensional materials provide aqueous environment in their pores favoring the biomolecule conformation preservation.

There are two strategies for immobilization of probes, namely physical adsorption and covalent binding. Covalent attachment is considered to be more preferable for immobilization of the probe since it guarantees a stable linkage with the support. In turn, a leakage of probe during washing steps is possible in the case of physical adsorption. As to covalent attachment, the functionality of solid support should provide the biomolecule immobilization under mild conditions to avoid the loss of its activity.

### 4.1. Protein Microarray

Protein microarray are highly demanded in various practical fields, such as molecular biology, medicine, analytical biotechnology, pharmacology, ecology, and bioinformatics [105,106,107]. Unlike DNA, proteins are more liable molecules that can easily change their conformation and, as a result, lose the ability to bind to a complementary biomolecule. In particular, they can change conformation during the interaction with hydrophobic support or because of the loss of their hydrate envelope. Thus, the most optimal supports for protein analysis are the porous hydrophilic matrices providing the aqueous microenvironment for both immobilized and analyzed proteins. Currently, the supports based on hydrophilic gels as well as nitrocellulose membranes are widely used as base for protein microarrays [108,109,110]. In comparison with swelling hydrogel layers, the washing steps for nitrocellulose membranes and macroporous monoliths, having non-swelling open porous space, takes less time. Additionally, the continuous gel matrix creates the significant limitations for protein macromolecules with low diffusion coefficients that results in significant time of analytical operations. In turn, comparing to nitrocellulose membranes, polymer monoliths allow much wider reactive functionalities for covalent immobilization of biomolecules.

**Figure 9:**
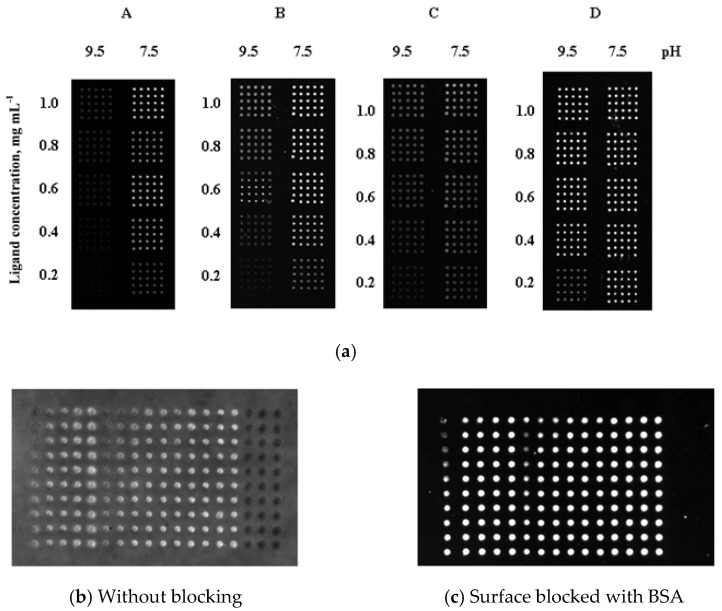
Dependence of analytical signal on pH of probe immobilization (**a**) ((**A**) poly(GMA-co-EDMA), (**B**) poly(GMA-co-GDMA), (**C**) poly(CEMA-co-HEMA-co-EDMA), (**D**) poly(CEMA-co-EDMA) layers) and effect of surface blocking (**b**,**c**) (poly(GMA-co-EDMA) layers). Reprinted with permission of Elsevier from [60].

The first polymer tested for microarray was poly(GMA-co-EDMA) [42]. This polymer is moderate in term of its hydrophobic-hydrophilic balance (contact angle 66.8 ± 0.3°) [53]. Moreover, the epoxide groups of this polymer allow the one-step covalent attachment of proteins to the polymer at mild conditions. Despite mentioned positive features, the kinetics of covalent attachment is quite slow and maximum of immobilization capacity is achieved within 16–20 h. It is known that reaction of amino groups with epoxides run more efficient at alkaline conditions (pH higher than 7.4). However, Rober et al. showed that analytical signal reflecting the efficiency of analyte to probe binding was much higher when immobilization of IgG as probe was performed at pH 7.4 (Figure 9a) [60]. Also, it was found that native poly(GMA-co-EDMA) possessed with autofluorescence which can be considerably reduced by surface blocking with bovine serum albumin (BSA) to reach high values of signal-to-noise (SNR) ratio (Figure 9b,c) [60].

To reduce the time of probe immobilization, Slabospitskaya et al. synthesized the new monolithic layers based on the terpolymer of N-hydroxyphthalimide acrylic acid ester (HPIEAA), GMA, and EDMA (poly(HPIEAA-co-GMA-co-EDMA)) and tested them for protein microarray [49]. The presence of highly reactive activated ester groups allows the immobilization of IgG within 2 h in phosphate buffer solution with pH 7.4. The immobilized probe bound the analyte with efficiency comparable to the same probe immobilized for 20 h on poly(GMA-co-EDMA) supports.

Furthermore, it was demonstrated that hydrophilization of polymer support due to the introduction of HEMA or replacement of EDMA with GDMA or DEGDMA led to the improvement of analytical potential of protein microarray based on macroporous monolithic layers in comparison to poly(GMA-co-EDMA) [55,61,64]. The positive effect on hydrophilization is connected with better surface wetting and penetration of biomolecules into the three-dimensional porous structure as well as with better preservation of active protein conformation. Moreover, the comparison of developed materials with 2-D glass-based microarrays revealed the dominance of macroporous monolithic layers in regards of analysis efficiency for the same protein affinity pairs [60,66].

In the paper published by Volokitina’s et al., the applicability of microarrays based on macroporous polymer monoliths for determination of quantitative parameters of affinity binding, namely the apparent dissociation constant of a specific complex and the maximum amount of bound analyte, has been shown [53]. The calculated dissociation constants for the affinity pair antigen-antibody were close to the known values for this kind of affinity interactions. The maximum amount of bound analyte increased with the growth of probe amount spotted onto the support surface.

As a practical example, the detection of a bone tissue marker, namely osteopontin, was developed using the macroporous poly(GMA-co-EDMA)-based layers [60]. The application of “sandwich” approach provided the detection limit of 0.3 pmol of protein/mL of solution. Also, the test-system for detection of antibodies to *Treponema pallidum* antigen, which causes syphilis, was developed using poly(GMA-co-EDMA) as support [111]. At the optimized analytical conditions, the sensitivity was found to be 92% that is close to the conventional immunoenzymatic assay (93.5%) while the amount of required biomaterial is about 100 times higher in the case of ELISA.

### 4.2. DNA Microarray

Besides analysis of proteins, macroporous monolithic layers appeared to be useful for the detection of DNA. In particular, Sinitsyna et al. demonstrated the efficiency of macroporous polymethacrylate layers as a new type of platforms for DNA microarray [112]. Five macroporous polymer monolithic materials with different hydrophilic-hydrophobic properties and reactive functionalities were tested for detection of cDNA (from *E. coli*) conjugated to a fluorescent Cy5-tag using the B2573RpoE oligonucleotide probe. Among the studied polymer layers, the hydrophilic poly(GMA-co-GDMA) and poly(HEMA-co-GDMA) supports demonstrated the best analytical potential. Nevertheless, the comparison of the results obtained with the application of macroporous platforms to those for widely used aldehyde glass slides confirmed the analytical surpassing of monolithic layers. As a practical example, the authors demonstrated the possibility to detect cystic fibrosis [112]. It was shown that macroporous monolithic layers can reliably distinguish mutated (positive) or healthy (negative) DNA fragment of gene material.

The test-system for detection of the most frequent variants of nucleotide substitutions in the human genome for the accurate diagnosis of mutations and polymorphisms of genes for pregnancy complications associated with thrombophilia were developed by Glotov et al. [65]. The correct detection of the polymorphisms in eight genes [F5 (Leiden G/A, rs6025); MTHFR (C/T, rs1801133); ITGB3 (T/C, rs5918); COMT (C/G, rs4818); TPH2 (T/A, rs11178997); PON1 (T/A rs854560); AGTR2 (C/A, rs11091046); and SERPINE1 (5G/4G, rs1799889)] was performed using microarray based on macroporous poly(GMA-co-EDMA) monolithic layers. Both the sensitivity and specificity of test-system were 98.6%. The comparison of the developed microarray to the standard NGS method demonstrated greater accuracy and specificity of monolithic layers for the case of nucleotide insertion/deletion changes.

### 4.3. Detection of Virus-Mimicking Particles and Viruses

The intraporous space of polymer monoliths is accessible not only for large macromolecules but also for virus nanoparticles [113]. This important property was utilized in the development of microarrays for virus detection. Kalashnikova et al. have designed the virus-mimicking particles (VMPs) based on polystyrene nanoparticles (NPs) of 80 nm covered with model protein (trypsin or transthyretin) [42]. The analysis of affinity binding between VMPs and complementary probe (soybean trypsin inhibitor or monoclonal antibody) was carried by two different methods: (1) direct fluorescent analysis and (2) sandwich enzyme-linked immunoassay. Poly(GMA-co-EDMA) layers were used as microarray support. The fluorescent method was able to sense the amounts up to 0.04–0.30 pmol/spot while immunoassay allowed the detection of 0.003–0.012 pmol/spot regarding to protein immobilized on the surface of NPs. It is worth noting that the apparent dissociation constant calculated from the non-flow-through microarray were rather close to those established by affinity chromatography. Further, the developed approach was applied for the creation of microarray for the analysis of influenza A and B viruses in biological samples. The sensitivity of the detection of influenza A and B viruses on a biochip was 93 and 80%, whereas the specificity was 61 and 83%, respectively [111].

Another practical application of monolithic-based microarray was their application for the detection of the E2 protein and hepatitis C virus-mimicking nanoparticles. The applicability of poly(GMA-co-DEGDMA) layers with different pore size as microarray platforms for direct and “sandwich” protocols based on binding of VMPs to immobilized anti-E2 antibodies or recombinant CD81-LEL was testified [114]. The identical detection efficiency of VMPs in a model buffer medium and spiked human blood plasma has proven the suitability of discussed materials for analysis in real biological fluids.

### 4.4. Determination of Enzyme Activity on a Chip

A method for simultaneous detection of acetylcholinesterase (AChE) and its activity using monolithic-based microarray has been recently developed by Volokitina et al. [53]. Anti-AChE antibodies were used as probe capable of selective binding of AChE. The detection of AChE was carried out via the use of non-fluorescent substrate, namely fluorescein acetate, which were hydrolyzed by AChE into fluorescent product. Using this substrate, a technique for the determination of activity of affinity bound AChE on a poly(GMA-co-DEGDMA) layers was developed. The catalytic activity of AChE determined on chip in buffer solution and human blood plasma were similar but twice lower than for in solution reaction. The slight lowering in AChE activity is explained by the partial changes in the conformation of enzyme active center related to the binding of AChE to immobilized antibody.

### 4.5. Molecularly Imprinted Microarray

In contrast to other supports applied for microarray (modified glass, nitrocellulose membranes, or polymer gels) and suitable for physical or covalent immobilization of a probe with further analysis of biomacromolecules, macroporous polymer monoliths can be prepared using molecular imprinting technique for both biomacromolecules and low molecular compounds [115]. The molecular imprinting method is based on the formation of recognition sites during the polymerization of functional and cross-linking monomers in presence of a template molecule. After the template removal, the prepared molecularly imprinted polymers (MIP) are characterized with a high specific recognition ability to the target molecule. To date, molecularly imprinted macroporous monoliths have been extensively studied in the various dynamic processes [116]. In particular, the effectiveness of MIP monoliths was confirmed in such methods as solid-phase extraction, HPLC and capillary electrochromatography for the isolation and analysis of both low-molecular objects [117] and proteins [118]. The most important issues to obtain the highly selective recognition centers using the molecular imprinting method are the correct choice of monomers, that ensure the formation of the pre-polymerization complex, and the fixation of the template imprint shape in the polymer matrix due to extensive crosslinking [119,120]. In the latter case, macroporous monolithic substrates are superior to gel-like polymer substrates, where the degree of crosslinking is quite low.

Recently, Antipchik et al. reported the preparation of macroporous molecularly imprinted monoliths for detection of low-molecular compounds in microarray format [73]. A set of macroporous poly(AEMA-co-HEMA-co-EDMA) layers containing the imprint sites of BOC-L-phenylalanine was prepared using non-covalent imprinting technique. Contrary to traditional microarrays, where the biological probes are immobilized on the solid support at spots, in the MIP monoliths all surface was capable of molecular recognition. Thus, the analysis was performed in the reversed way when analyte solutions were spotted onto the surface. The good analyte rebinding from buffer solution with recognition factors 2.5–3.4 was demonstrated. Moreover, the comparable rebinding efficiency was detected for the analysis in the different biological media (simulated blood plasma and human blood plasma). The selectivity of binding from the equimolar mixture of L-phenylalanine and L-tyrosine, which are structure analogues, was 81.9% for free L-phenylalanine and 91.2% for the FITC-labeled L-phenylalanine.

## 5. Concluding Remarks

The summarized in this review data confirm the successful applicability of polymer monolithic sorbents as the stationary phases not only in well-known and widely accepted flow-through techniques but also in thin layer format. The simplicity of layer formation via in situ polymerization represents the great adventure of such devices. Hydrophobic-hydrophilic properties as well as chemical reactivity of polymer material can be easily changed by variation of functional monomer(s) and crosslinker as well as by the surface post-modification. The remaining deficiency is to obtain reproducible polymer layers, since the reproducibility of the synthesis directly determines the reproducibility of the analysis. This problem can be overcome by optimizing technical solutions in obtaining molds for polymerization.

The presented results of TLC clearly show that open and accessible for any molecular sizes porous structure allows avoiding the restrictions of diffusively controlled mechanism of mass transfer usually observed for particle-based separation media. As a rule, the high speed of separation process was demonstrated. Additionally, the nature of functional monomer(s) gives a possibility to realize different mechanism of separation. Taking into account the possibility of a wide adjustment of the surface chemistry, in addition to various types of one- and two-dimensional planar chromatography techniques, macroporous monolithic layers can also be successfully used for planar electrochromatography.

The application of polymer monoliths as platforms for microarray has demonstrated high sensitivity and specificity of analysis on biochip. Simple production, diversity of monomers providing the reactive functionality and hydrophilic/hydrophobic properties, high surface capacity as well as porous structure stable in dry and wet states are undoubtedly the advantages of these materials compared to other carriers used in the microarray format. The unique properties of macroporous polymer monoliths make this type of materials universal in the bioanalysis of objects of various nature such as proteins, nucleic acids, and viruses. In addition, the possibility of obtaining molecularly imprinted monolithic layers allows for microarray analysis of low-molecular weight metabolites in biological fluids. Thus, polymer monoliths have significant perspectives for the application in discussed scientific and practical fields.

## Figures and Tables

**Table 1:** Summary on preparation and application of macroporous monolithic polymer layers.

Supporting Surface and Its Treatment	Monomers	Porogens	Initiator and Conditions of Polymerization	Polymerization Approach and Technical Implementation	Modification/ Application	Ref.
7.6 × 2.6 cm glass slide; silanization with TMSPMA	BMA and EDMA	1-decanol,cyclohexanol	2,2-dimethoxy-2-phenylacetophenone; UV-polymerization (254 nm), 15 min	Free radical polymerization in a mold consisting of two glass plates clamped together and separated with Teflon strips. Polymer thickness was varied from 50–200 µm	TLC separation of peptides and proteins with MALDI-TOF-MS detection	[41]
12.0 × 3.3 cm glass plate; silanization with TMSPMA	GMA and EDMA	1-decanol,cyclohexanol	2,2-dimethoxy-2-phenylacetophenone; UV-polymerization (254 nm), 15 min	Free radical polymerization in a mold consisting of two glass plates clamped together and separated with Teflon strips. Polymer thickness was 50 µm	Photografted with LMA and PEGMA; 2-D TLC separation of peptides with MALDI-TOF-MS detection	[56]
12.0 × 3.3 cm glass plate; silanization with TMSPMA	BMA and EDMA	1-decanol, cyclohexanol	2,2-dimethoxy-2-phenylacetophenone; UV-polymerization (254 nm), 15 min	Free radical polymerization in a mold consisting of two glass plates clamped together and separated with Teflon strips. Polymer thickness was 50 µm.	Photografted with AMMPSA and EDMA; 2-D TLC separation of peptides with DESI-MS detection; ElectroTLC separation of peptides.	[50,62]
12.0 × 3.3 cm glass plate; silanization with TMSPMA	BMA and EDMA; HEMA and EDMA; ST and DVB	1-decanol/ 1-dodecanol cyclohexanol	(a) 2,2-dimethoxy-2-phenylacetophenone; UV-polymerization (254 nm), 15 min; (b) AIBN; 70 °C, 24 h	Free radical polymerization in a mold consisting of two glass plates clamped together and separated with Teflon strips. Polymer thickness was 50 µm	Photografted with PFPMA	[55]
6.0 × 3.3 cm glass plate; silanization with TMSPMA	MST, CHMST and DVB	toluene, 1-dodecanol	AIBN; 70 °C, 20 h	Free radical polymerization in a mold consisting of two glass plates clamped together and separated with Teflon strips. Polymer thickness was 50 µm	After polymerization the hyper-crosslinking reaction was carried out in 1,2-dichloroethane in a beaker for 2 h; TLC separation of peptides with MALDI-TOF-MS.	[57]
N/A	GMA and EDMA; BMA and EDMA	1,4-butandiol,cyclohexanol	2-methoxy-2phenylacetophenine; 2-methyl-propiophenone; UV- polymerization (254 nm), 30–50 min	Free radical polymerization in a mold consisting of two glass plates clamped together and separated with Teflon strips. Polymerization of two monomer mixtures through the mask separately; Polymer thickness was 200 µm	Hydrolysis of poly(GMA-co-EDMA) area; 2-D TLC separation of dyes with SERS-detection	[51]
N/A	GMA and EDMA	1-decanol, cyclohexanol	2,2-dimethoxy-2-phenylacetophenone; UV-polymerization	N/A	Functionalized via a “thiol-ene” click reaction with mixture of 10-undecylenic acid and LMA.	[63]
7.5 × 2.5 cm glass slide; silanization with TMSPMA	GMA and EDMA; BMA and EDMA; AEMA, HEMA and EDMA; CEMA, HEMA and EDMA	1-decanol, cyclohexanol,1,4-butandiol,NMP, DMF, PEG-200	2-hydroxy-2-methylpropiophenone; UV-polymerization (wide spectrum), 20–30 min	Free radical polymerization in a well 6 × 2 cm covered with quartz cap. Operating wells were prepared by glass etching with 11 M HF for 30 min. Polymer thickness was 200 µm	TLC separation of dyes, DNP-amino acids and synthetic polymers.	[52]
Glass slide	ST, BA and DVB	1 wt% CaCl_2_ aqueous solution	benzoyl peroxide; 70 °C, 8 h	High internal phase emulsion polymerization in mold formed on the surface of glass slide.	TLC separation of components of Chinese herbs	[48]
6.6 × 3.3 cm glass plate; silanization with TMSPMA	GMA and EDMA	1-decanol, cyclohexanol	2,2-dimethoxy-2-phenylacetophenone; UV-polymerization (365 nm), 15 min	Free radical polymerization in mold consisting of two glass plates clamped together and separated with Teflon strips.	Surface modification with gold nanoparticles; SERS-detection of bacteria.	[64]
7.5 × 2.5 cm glass slide; silanization with TMSPMA	GMA and EDMA	cyclohexanol	2-hydroxy-2-methylpropiophenone; UV-polymerization (wide spectrum), 20 min	Free radical polymerization in a well 6.0 × 1.8 cm covered with quartz cap. Operating wells were prepared by glass etching with 11 M HF for 30 min. Polymer thickness was 200 µm.	Analysis of virus-mimicking particles and DNA in microarray format.	[42,65]
7.5 × 2.5 cm glass slide; silanization with TMSPMA	HEMA and GDMA	polystyrene in toluene, 1-decanol, cyclohexanol	benzophenone, benzoin methyl ether, 2-hydroxy-2-methylpropiophenone; UV-polymerization (wide spectrum)	Free radical polymerization in a well 6.0 × 2.0 cm covered with quartz cap. Operating wells were prepared by glass etching with 11 M HF for 30 min. Polymer thickness was 200 µm	Different protein immobilization techniques; analysis of proteins in microarray format.	[66]
7.5 × 2.5 cm glass slide; silanization with TMSPMA	GMA and GDMA; CEMA and GDMA; CEMA, HEMA and EDMA; HPIEAA, GMA and EDMA; GMA and DEGDMA	1-decanol, toluene, heptane, cyclohexanol,PEG-200-600	2-hydroxy-2-methylpropiophenone; UV-polymerization (wide spectrum), 20–30 min	Free radical polymerization in a well 6.0 × 2.0 cm covered with quartz cap. Operating wells were prepared by glass etching with 11 M HF for 30 min. Polymer thickness was 200 µm	Analysis of DNA, proteins, virus-mimicking particles in microarray format.	[54,55,61, 65, 66, 67]

Abbreviations: TMSPMA: 3-(trimethoxysilyl)propyl methacrylate; GMA: glycidyl methacrylate; EDMA: ethylene dimethacrylate; BMA: butyl methacrylate; LMA: lauryl methacrylate; PEGMA: poly(ethylene glycol) methacrylate; AMMPSA: 2-acrylamido-2-methyl-1-propanesulfonic acid; ST: styrene; DVB: divinylbenzene; MAOPfp: pentafluorphenyl ester of acrylic acid; MST: methylstyrene; CHMST: chloromethylstyrene; HEMA: 2-hydroxyethyl methacrylate; AEMA: 2-aminoethyl methacrylate; CEMA: 2-cyanoethyl methacrylate; GDMA: glycerol dimethacrylate; HPIEAA: N-hydroxyphtaleimide ester of acrylic acid; DEGDMA: di(ethylene glycol) dimethacrylate; PFPMA: 2,2,3,3,3-pentafluoropropyl methacrylate; DMF: dimethylformamide; NMP: N-methylpirrolidone; MALDI-TOF-MS: matrix-assisted laser desorption/ionization-time of flight mass-spectrometry; SERS: surface-enhanced Raman spectroscopy.

## Data Availability

Not applicable.
